# Formononetin, a Key Component of Danggui Buxue Decoction, Inhibits Metastasis of Triple‐Negative Breast Cancer by Modulating SNAI2‐Driven EMT

**DOI:** 10.1002/fsn3.71286

**Published:** 2025-12-04

**Authors:** Kui Li, Ting Zhou, Ruoxia Wu, Jiaqing Xiong

**Affiliations:** ^1^ Department of Anorectal Surgery The First Hospital of Hunan University of Chinese Medicine Changsha China; ^2^ Department of Clinical Chinese Pharmacy, College of Pharmacy Hunan University of Chinese Medicine Changsha China; ^3^ College of Traditional Chinese Medicine Hunan University of Chinese Medicine Changsha China; ^4^ Department of Mammary The First Hospital of Hunan University of Chinese Medicine Changsha China

**Keywords:** Danggui Buxue Decoction, metastasis, SNAI2, triple‐negative breast cancer

## Abstract

Danggui Buxue Decoction (DBD) has shown potential antitumor effects in a variety of cancers. This study aims to delve into the effects and mechanisms of DBD on the metastasis of triple‐negative breast cancer (TNBC). The mouse model of TNBC was constructed, and the function of DBD was investigated. The active components and core targets of DBD on TNBC were identified through liquid chromatography‐mass spectrometry, network pharmacology, and molecular docking techniques. A set of in vitro experiments was conducted to examine the key components and core targets of DBD that affect the metastasis of TNBC. DBD inhibited the growth and metastasis of TNBC tumors. Formononetin is one of the key active ingredients of DBD, exerts antitumor effects by targeting Snail family transcriptional repressor 2 (SNAI2). The addition of DBD or Formononetin inhibited the malignant activity and reduced the expression of SNAI2. However, this ability of Formononetin was reversed due to the overexpression of the SNAI2 gene. Formononetin inhibits the metastasis of TNBC by downregulating SNAI2. The outcomes offer a basis for the future clinical utilization of DBD and Formononetin in TNBC therapy.

## Graphical abstract

Formononetin, the key active ingredient of Danggui Buxue Decoction, directly targets and downregulates the transcription factor SNAI2. This inhibition suppresses epithelial‐mesenchymal transition (EMT), thereby reducing the invasion and metastasis of triple‐negative breast cancer cells both in vitro and in vivo. Our findings elucidate a novel molecular mechanism by which Danggui Buxue Decoction and its component Formononetin exert antitumor effects.

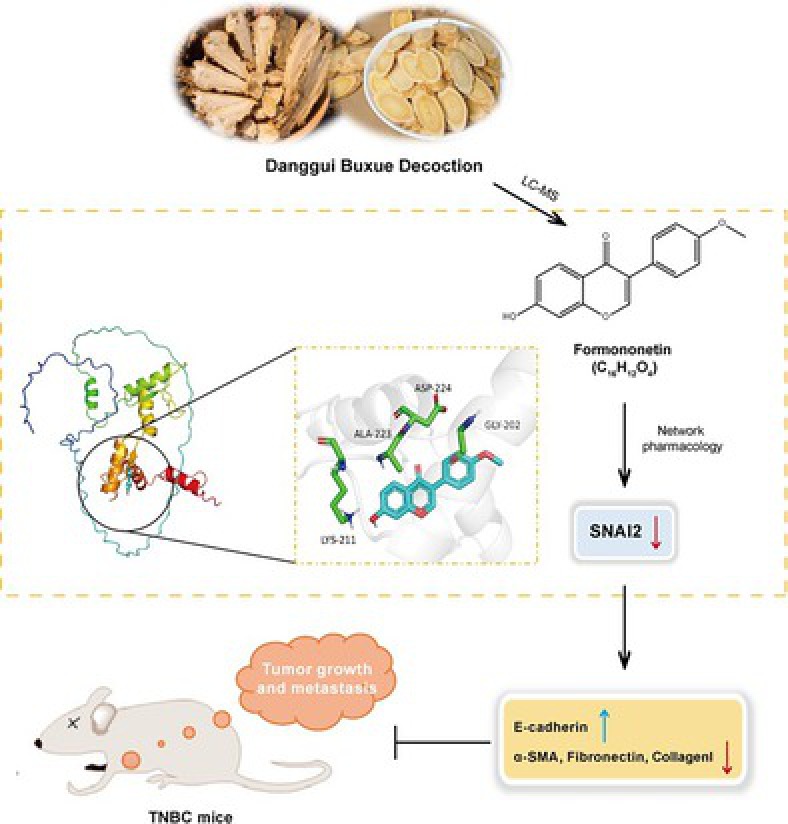

## 1 Introduction

Among women around the world, breast cancer is a primary cause of death related to cancer (Arnold et al. 2022; Sung et al. 2021). Triple‐negative BC (TNBC) is one of its main subtypes (Garrido‐Castro et al. 2019). Statistical analysis indicates that 30%–40% of TNBC patients relapse within 5 years (Leon‐Ferre and Goetz 2023).

Recent studies highlight the potential of Traditional Chinese Medicine (TCM) in cancer therapy, particularly its immunomodulatory and antitumor properties (Bao et al. 2023; Qi et al. 2015; Yuan et al. 2024). Danggui Buxue Decoction (DBD), a TCM formula comprising *Astragalus membranaceus (Fisch.) Bunge* and *Angelica sinensis (Oliv.) Diels* contains a multitude of active ingredients, including saponins, flavonoids, and polysaccharides, despite its relatively simple composition (Jiang et al. 2023). In non‐small cell lung cancer studies, DBD has been shown to inhibit tumor growth (Zhu et al. 2024). DBD suppresses abnormal red cell progenitor accumulation in melanoma xenograft rats and inhibits tumor growth and metastasis (Li et al. 2019). Nevertheless, the active components and targets of DBD in the treatment of TNBC have yet to be fully elucidated.

Currently, it is widely believed that epithelial‐mesenchymal transition (EMT) can drive the metastasis of various tumors, including TNBC (Grasset et al. 2022; Jiang et al. 2022). Previous research has highlighted snail family transcriptional repressor 2 (SNAI2) as a crucial molecule involved in EMT in BC (Li, Deng, et al. 2023). Increased levels of SNAI2 have been shown to enhance the spread of BC (Li et al. 2020). Moreover, studies have indicated that the Ruyiping formula exerts antimetastatic activity by targeting miR‐134 to inhibit SNAI2 (Jiang et al. 2021). *Polygonum multiflorum* can inhibit the expression of SNAI2, thus inhibiting the spread of bladder cancer (Liu et al. 2020). While some studies have investigated the role of TCM in inhibiting tumor spread by reducing SNAI2 expression, the mechanistic exploration of how DBD and its bioactive compounds modulate SNAI2 expression to inhibit BC progression remains underexplored.

To sum up, this study aims to identify the essential compound components and target mechanisms of DBD in anti‐TNBC through liquid chromatography‐mass spectrometry, network pharmacology, and cell and animal experiments. Notably, by elucidating DBD's multi‐target synergistic effects, this research seeks to establish a scientific rationale for its application as an adjuvant therapy alongside conventional treatments (e.g., chemotherapy and immunotherapy), thus offering improved direction for choosing TCM adjuvant treatment strategies in clinical settings.

## 2 Materials and Methods

### 2.1 Preparation of DBD

A ratio of 5:1 (dry weight) of *Astragalus membranaceus (Fisch.) Bunge* and *Angelica sinensis (Oliv.) Diels* sinensis was mixed, soaked in eight times its volume of distilled water for 0.5 h. Then, it was centrifuged at 5000 rpm/min for 10 min to obtain the supernatant. Following this, the drug residue was combined with distilled water in a volume five times greater, and the previous steps were repeated. The supernatant was frozen overnight in a −80°C refrigerator. It was concentrated to 0.6 g/mL as a freeze‐dried powder using a freeze dryer (Eyel4 model freeze dryer, Tokyo, Japan), and stored at 4°C before the experiment (Feng et al. 2021).

### 2.2 Cell Culture

DMEM/F12 medium (AW‐M006, Abiowell, China) was used as a culture medium for MDA‐MB‐231 (AW‐CCH048) and MCF‐10A (AW‐CNH264). MDA‐MB‐468 (AW‐CCH272) was cultured in Leibovitz's L‐15 medium (AW‐M009). Both media had 10% FBS (10,099,141, Gibco) and 1% Penicillin–Streptomycin solution (AWH0529, Abiowell). All cells (Abiowell) were incubated in a 37°C, 5% CO_2_ cell culture incubator.

### 2.3 TNBC Mouse Model

To create the TNBC mouse model, MDA‐MB‐231 cells (5 × 10^6^) were injected subcutaneously into the right axilla (fourth mammary pad) of the BALB/c nude mice (6–8 weeks, Hunan SJA) (Song et al. 2020; Verma et al. 2019). The drug treatment began once the tumor size was about 100 mm^3^.

The mice were randomly divided into the Model group, Model + DBD group, and Model + Paclitaxel group (6 mice per group). The Model group was established using the methodology described above and was given normal saline. The Model + DBD group was given DBD intragastrically at a daily dose of 7.2 g/kg. (Nair and Jacob 2016; Xu et al. 2023). In the Model + Paclitaxel group, the model group mice were given oral Paclitaxel at a dose of 50 mg/mouse as a positive control for treatment (Li et al. 2021). The growth status was observed daily, and tumor volume was monitored every 4 days throughout the treatment period. Tumor volume = 1/2 × (length × width × width). After 4 weeks, the tumors were excised, photographed, and subjected to further analysis. The First Hospital of Hunan University of Chinese Medicine's Animal Ethics Committee approved the animal experiment (No. 2023‐014).

### 2.4 Immunohistochemistry Staining

Following established protocols (Pan et al. 2020), immunohistochemical staining was performed. Tumor tissue paraffin sections were baked in a 60°C incubator for 12 h. Then, after heat‐induced antigen retrieval, the sections were incubated with an anti‐Ki67 (27309–1‐AP, 1:5000, Proteintech, USA) antibody. Sections were observed under an optical microscope (Motic) after staining with DAB (ZLI‐9018, Jinqiao, Zhongshan, China).

### 2.5 Biological Safety Assessment and Histopathological Morphology

Formononetin (50 mg/kg) was administered to normal mice via gavage and the in vivo biological safety was evaluated using the hematoxylin–eosin (H&E) staining.

In accordance with standard procedures (Feng et al. 2021), staining with H&E was conducted on the liver, kidney, or tumor paraffin sections (AWI0001a and AWI0029a, Abiowell). Subsequently, images were obtained using a light microscope (BA210T, Motic, China).

### 2.6 High‐Performance Liquid Chromatography–Tandem Mass Spectrometry (HPLC‐MS) Analysis

Chromatographic separation was achieved using a Thermo (USA) C18 column (150 × 4.6 mm, 5 μm). A solution of water with 0.1% formic acid (phase A) and acetonitrile (phase B), flowing at 0.2 mL/min, with an injection volume of 10 μL. Gradient elution procedure: 90% A ~ 50% A, 0 ~ 4 min. 50% A ~ 40% A, 4 ~ 15 min. 40% A ~ 20% A, 15 ~ 18 min, 20% A ~ 90% A. 18 ~ 19 min. 90% A, 19 ~ 25 min. Electrospray ionization in both positive and negative ion modes was employed to examine DBD samples. For the mass spectrometer, the source temperature is 325°C, the gas 1 and gas 2 are maintained at 55 psi each, the curtain gas is set at 35 psi, and the capillary voltage is 4.5 kV. Data were acquired in MRM mode by keeping track of chosen ions.

### 2.7 Target Analysis of Formononetin and TNBC

Formononetin targets were sought in the GeneCards database, with targets from the Swisstargets database selected based on a probability score > 0. The obtained targets were then subjected to further standardized and deduplication using the UniProt database.

A search for “TNBC” was conducted on the GeneCards database, the NCBI database, and the CTD database. The targets obtained from the CTD database were filtered based on median scores to obtain a more relevant set of targets. In order to generate a list of TNBC‐related target genes, the targets from the three databases were merged and de‐duplicated.

### 2.8 Protein–Protein Interaction (PPI) Network Construction

Formononetin and TNBC had their shared *Homo sapiens* targets constructed using the STRING database. Core genes were selected using MCODE analysis. The interactions involving Formononetin, TNBC, and their targets were used to create a network diagram with Cytoscape software.

### 2.9 GO and KEGG Pathway Analyses

Based on Metscape, GO enrichment analysis was performed on shared targets between Formononetin and TNBC (https://metascape.org/gp/index.html). The filtering criterion was a corrected *p*‐value < 0.05.

### 2.10 Molecular Docking Analysis

PubChem was the source for the 3D model of Formononetin. Protein targets' three‐dimensional structures were obtained from RCSB PDB. Molecular docking analysis was conducted using AutoDock Vina software to analyze the interaction between protein receptors and active compounds preliminarily. The binding energy of docking was calculated, and docking heat maps were generated using subsequent molecular docking simulations.

### 2.11 Drug Treatment In Vitro

Cells were treated with either DBD (0, 0.125, 0.25, 0.5, and 1 mg/mL) or Formononetin (0, 10, 20, 40, and 80 μmol/L) as single agents for 48 h. This quantified cell viability by CCK8 to determine optimal treatment concentrations. The OD was measured at 450 nm (MB‐580, Hui Song, China).

### 2.12 Cell Transfection

The overexpression plasmids for SNAI2 (oe‐SNAI2) and control (oe‐NC) were obtained from HonorGene (China). The oe‐SNAI2 plasmid was transfected into cells using a transfection reagent (11668‐019, Invitrogen, USA) for 48 h.

### 2.13 Wound Healing Assay

5 × 10^5^ cells/well were seeded in 6‐well plates, a scratch was created at the bottom of each well with a 200 μL pipette tip once the cells reached 80%–90% confluence. The dislodged cells were washed off with sterile PBS and maintained in a serum‐free medium. The images were observed under a microscope at 0 and 24 h after scratching.

### 2.14 Transwell Invasion Assay

The Matrigel matrix gel‐coated (3428, Corning, USA) Transwell chambers received 100 μL of the 2 × 10^6^ cell suspension. The lower chamber received 500 μL of medium with FBS. After a 48‐h incubation period at 37°C, the chambers were removed. To fix them, the cells were exposed to 4% paraformaldehyde (AWI0056b, Abiowell) for 20 min. The cells were observed under a microscope after being stained with 0.1% crystal violet (AWC0333a, Abiowell).

### 2.15 Quantitative Reverse Transcription Polymerase Chain Reaction (qRT‐PCR)

With the TRIzol reagent kit, total RNA was extracted from both tissue and cell samples (15596026, Thermo, USA). Using the HiFiScript cDNA Synthesis Kit, the extracted RNA was reverse‐transcribed into cDNA, and a fluorescence quantitative PCR reaction was performed using the UltraSYBR Mixture. The data were analyzed using the 2^−ΔΔ*C*t^ method, with normalization to β‐actin (Table 1).

**TABLE 1:** Primers amplified by qRT‐PCR.

Name of primer	Sequences	Product length (bp)
H‐SNAI2‐F	AGACCCCCATGCCATTGAAG	79
H‐SNAI2‐R	GGCCAGCCCAGAAAAAGTTG	
H‐β‐Actin‐F	ACCCTGAAGTACCCCATCGAG	224
H‐β‐Actin‐R	AGCACAGCCTGGATAGCAAC	
M‐β‐Actin‐F	ACATCCGTAAAGACCTCTATGCC	223
M‐β‐Actin‐R	TACTCCTGCTTGCTGATCCAC	

### 2.16 Western Blot

Total protein was quantified using a BCA protein quantification kit (P0010S, Beyotime). The separated proteins by SDS‐PAGE were transferred to nitrocellulose membranes and blocked with 5% skimmed milk (Abiowell) for 90 min. The membranes were incubated with primary antibodies SNAI2 (12129‐1‐AP, 1:5000, Proteintech), E‐Cadherin (20874‐1‐AP, 1:20000, Proteintech), α‐SMA (14395‐1‐AP, 1:1000, Proteintech), Collagen I (ab138492, 1:100, Abcam), Fibronectin (15613‐1‐AP, 1:200, Proteintech), and β‐actin (AWA80001, 1:500, Abiowell) overnight at 4°C. After incubation with secondary antibodies, protein bands were visualized using ECL Plus Ultra‐sensitive luminescent liquid (AWB0005, Abiowell) and images were obtained with a gel imaging system (ChemiScope6100, Clinx, China).

### 2.17 Drug Affinity Responsiveness Target Stability (DARTS)

The cells (1 × 10^7^ cells/mL) were lysed using the RIPA lysis buffer to obtain a protein concentration of approximately 2 mg/mL (measured by the BCA assay). The obtained lysates were diluted with TNC buffer (50 mM Tris–HCl (pH 8.0), 50 mM NaCl, 10 mM CaCl_2_). Five portions of the lysates were prepared and exposed to DMSO or different doses of Formononetin (10, 20, and 40 μM) for 60 min. Then, 0.3 μg/mL of Pronase was added to induce limited proteolysis, which can break down unbound proteins while not disrupting the target proteins that are bound to the drug due to conformational protection. After 15 min, the protein of SNAI2 was detected.

### 2.18 AI Usage Statement

“No artificial intelligence tools were used in the process of data generation, analysis, or visualization. The AI‐assisted writing tool, such as Grammarly and ChatGPT, was only employed during the draft writing stage for grammar and style corrections.”

### 2.19 Data Analysis

All data were statistically analyzed using GraphPad Prism 8.0 software and presented as mean ± standard deviation (SD). Statistical analysis for intergroup differences was performed using one‐way or two‐way analysis of variance followed by a post hoc Tukey test. A *p*‐value < 0.05 was considered statistically significant.

## 3 Results

### 3.1 DBD Inhibits TNBC

**FIGURE 1:**
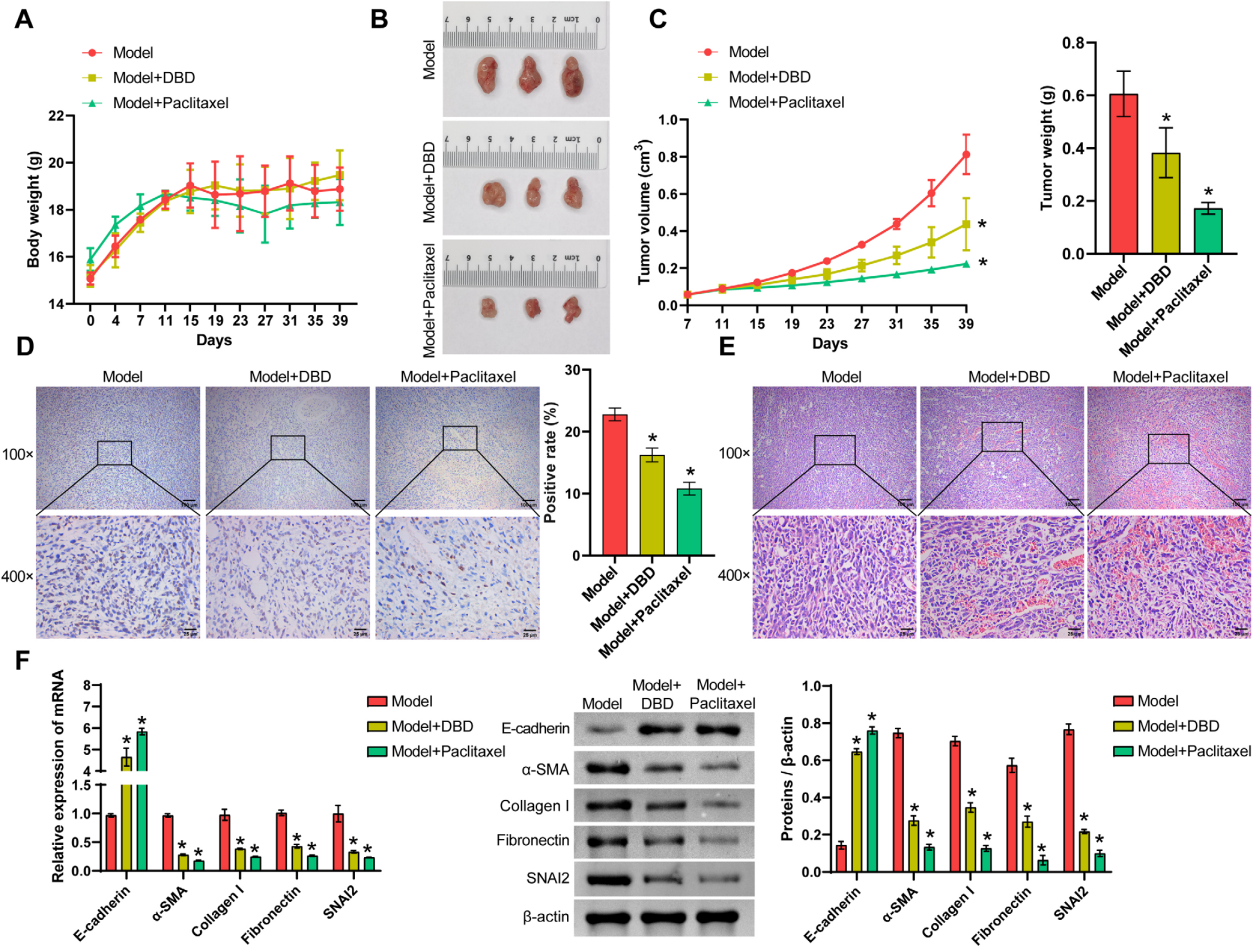
DBD inhibits TNBC tumor growth and metastasis. (A) Mouse weight. (B) Tumor body. (C) Tumor volume growth curve and weight. (D) The proportion of positive Ki‐67 expression. (E) Histopathological morphology of nude mice. (F) The expression of E‐cadherin, α‐SMA, Collagen I, Fibronectin, and SNAIL2. Data are presented as mean ± SD, *n* = 6, * vs. the Model group, *p* < 0.05. Model group: untreated TNBC tumor‐bearing mice. Model + DBD group: TNBC mice were treated with DBD. Model + Paclitaxel group: TNBC mice were treated with Paclitaxel.

The anti‐TNBC effects of DBD were investigated by inoculating MDA‐MB‐231 cells into the nude mice. The intervention treatment with DBD or Paclitaxel was initiated on the 11th day, when significant tumors were observed. As shown in Figure 1A, neither DBD nor Paclitaxel had a significant effect on the body weight of the mice. As illustrated in Figure 1B,C, tumor growth, volume, and weight were markedly reduced in the Model + DBD and Model + Paclitaxel groups relative to the Model group, with no discernible difference in mouse weight among the groups. Moreover, the immunohistochemistry staining results of Ki67 demonstrated that treatment with DBD could inhibit tumor proliferation (Figure 1D). As illustrated in Figure 1E, the Model + DBD and Model + Paclitaxel groups exhibited varying degrees of tumor cell necrosis. Notably, following treatment with DBD or Paclitaxel, the expression of α‐SMA, Collagen I, Fibronectin, and SNAIL2 in tumor tissues was significantly suppressed. In contrast, E‐cadherin expression was significantly increased (Figure 1F). These findings indicate that DBD is efficacious in inhibiting tumor growth and metastasis in vivo.

### 3.2 Formononetin Was Identified as a Principal Active Ingredient in DBD

**FIGURE 2:**
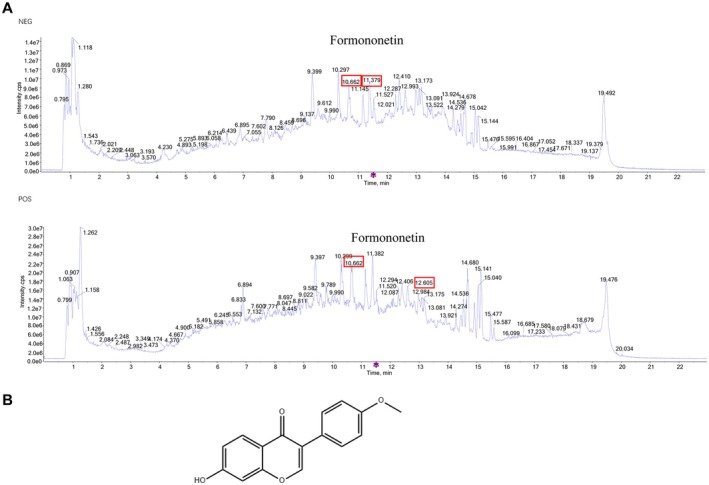
Formononetin was identified as the principal active ingredient in DBD. (A) HPLC‐MS analysis was used to demonstrate the presence of Formononetin as the principal active ingredient in DBD. (B) The chemical formula of Formononetin.

To ascertain the key active ingredients of DBD, we conducted an identification and analysis using HPLC‐MS. Consistent with previous reports (Feng et al. 2021), Formononetin was one of the key active ingredients in DBD (Figure 2A). The structure of Formononetin (C_16_H_12_O_4_) is depicted in Figure 2B.

### 3.3 Network Pharmacology Analysis of the Interaction Between Formononetin and the Targets Related to TNBC

**FIGURE 3:**
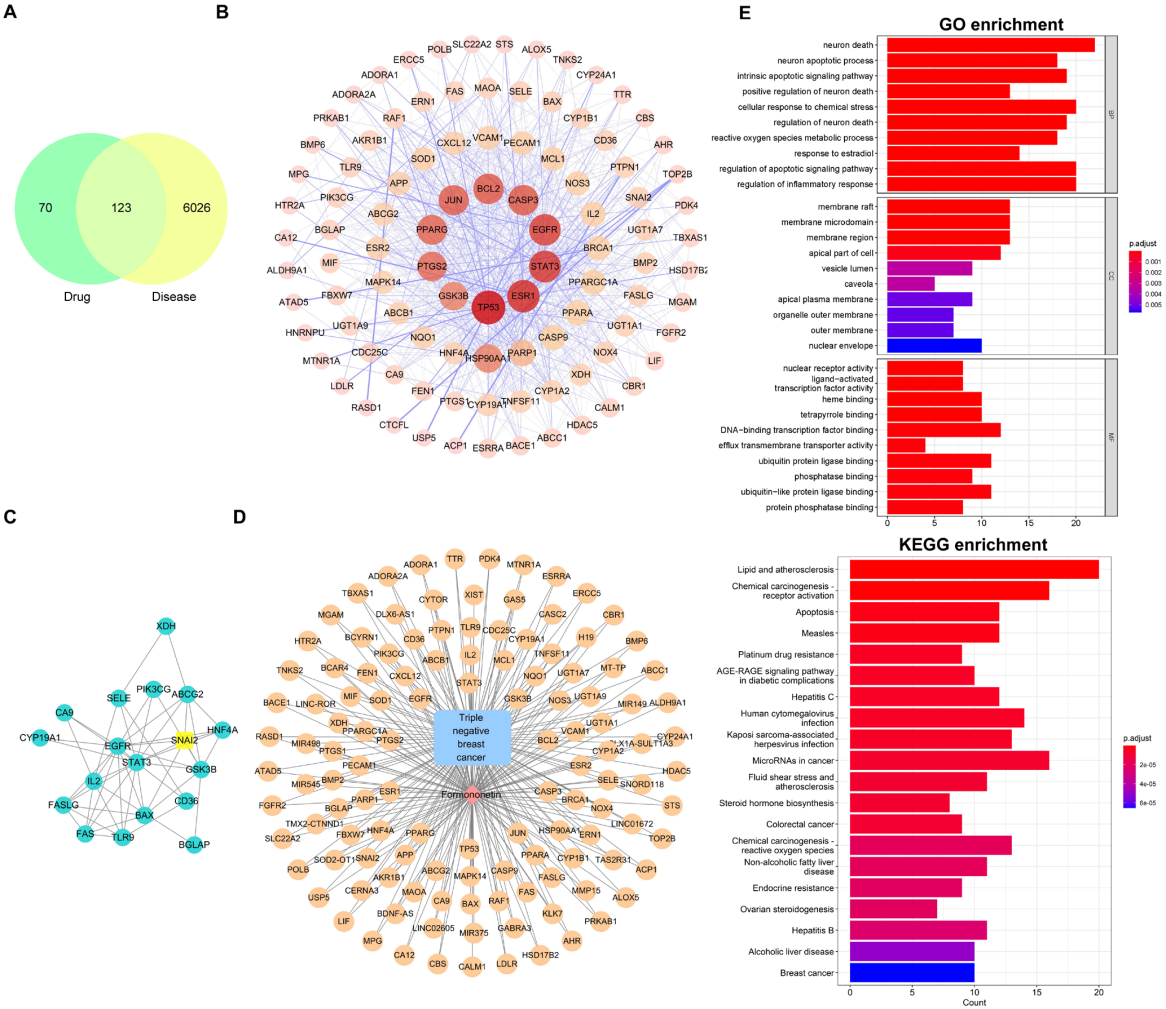
Network pharmacology analysis of key interactions. (A) Venn diagram illustrating the shared targets of the Formononetin and TNBC. (B) The identification of key proteins was based on a PPI network. (C) Cluster analysis of the core gene SNAI2. (D) Construction of a network to illustrate the interactions between the Formononetin–TNBC targets. (E) GO and KEGG enrichment analysis.

We then explored common targets for Formononetin and TBNC. A Venn diagram was constructed with the 6149 TNBC‐related genes and 193 Formononetin targets, indicating that 123 genes may be potential targets of Formononetin acting on TNBC, as shown in Figure 3A. The STRING online platform was used to import the 123 genes and construct the network diagram based on the Cytoscape software (Figure 3B). Following the application of the MODE clustering analysis, SNAI2 was identified as one of the core genes (Figure 3C).

As illustrated in Figure 3D, we constructed a network diagram representing the interactions between Formononetin and TNBC targets. Further, as shown in Figure 3E, several pathways, including lipid and atherosclerotic signaling pathways, apoptosis, and receptor activation, are involved in the Formononetin treatment of TNBC.

### 3.4 Formononetin Targeting SNAI2

**FIGURE 4:**
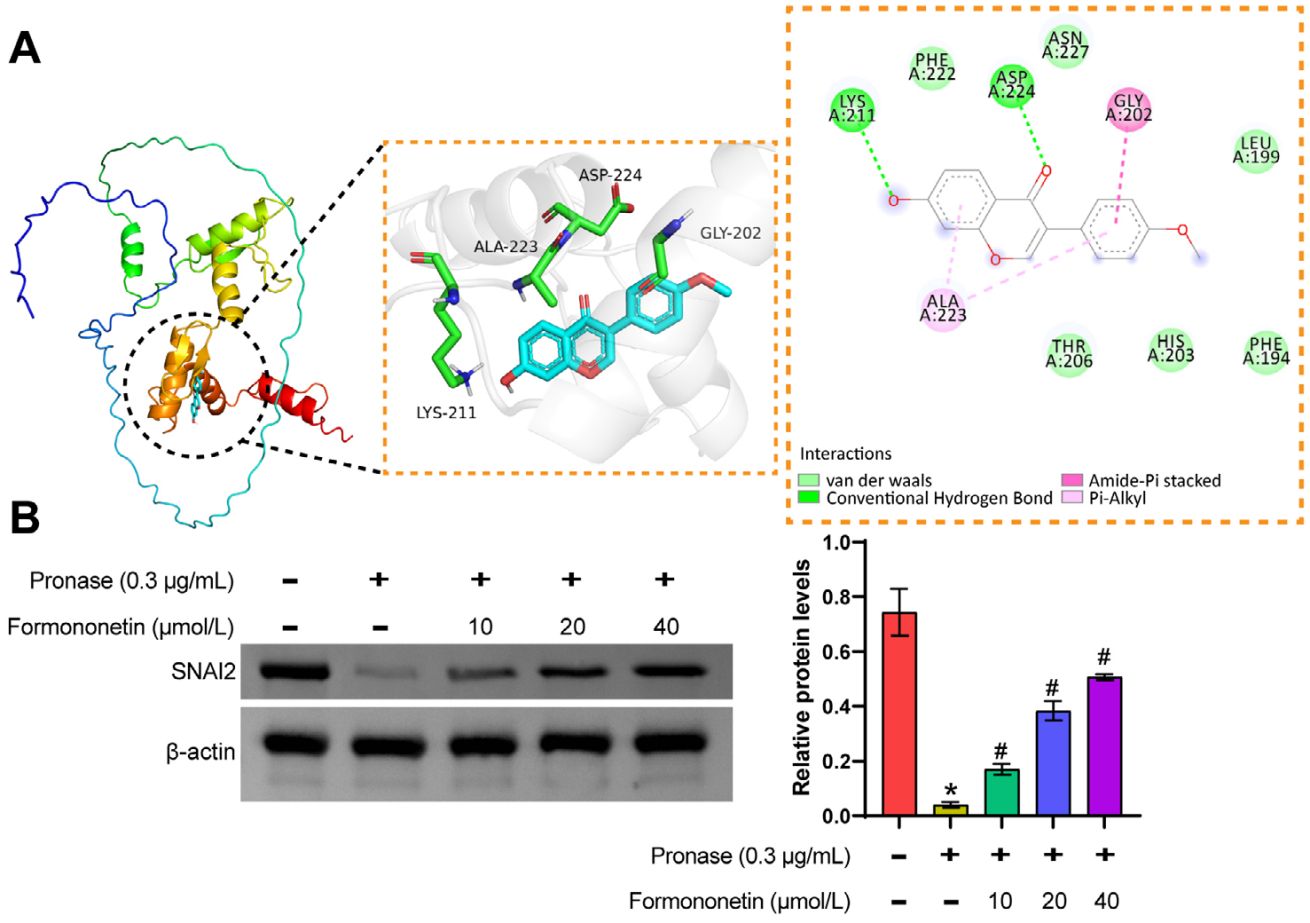
Formononetin targeting SNAI2. (A) Molecular docking analysis was used to predict the binding of the active ingredient Formononetin to the core gene SNAI2. (B) The binding of Formononetin to SNAI2 was confirmed by the DARTS method. Data are presented as mean ± SD, *n* = 3. *p < 0.05 vs. the Control group (0 μm/mL Formononetin + 0 μg/mL Pronase), #*p* < 0.05 vs. the Pronase group. Control group: Not treated with Pronase and Formononetin. Pronase group: Only treated with Pronase.

SNAI2 is recognized as a transcription factor associated with EMT and is crucial in tumor metastasis (Fan et al. 2024). Therefore, we performed molecular docking of the predicted active ingredient Formononetin with the core gene SNAI2. Molecular docking revealed a binding energy of −6.4 kcal/mol between Formononetin and SNAI2 (Figure 4A), indicating a potential strong binding affinity. Further, we confirmed the combination of Formononetin and SNAI2 by the DARTS method. In the co‐incubation system with Pronase, the higher the concentration of Formononetin, the greater the protection of SNAI2 protein from Pronase degradation (Figure 4B). This suggests that Formononetin may regulate TNBC metastasis by targeting SNAI2.

### 3.5 Formononetin Inhibits the Malignant Function of TNBC by Regulating SNAI2

**FIGURE 5:**
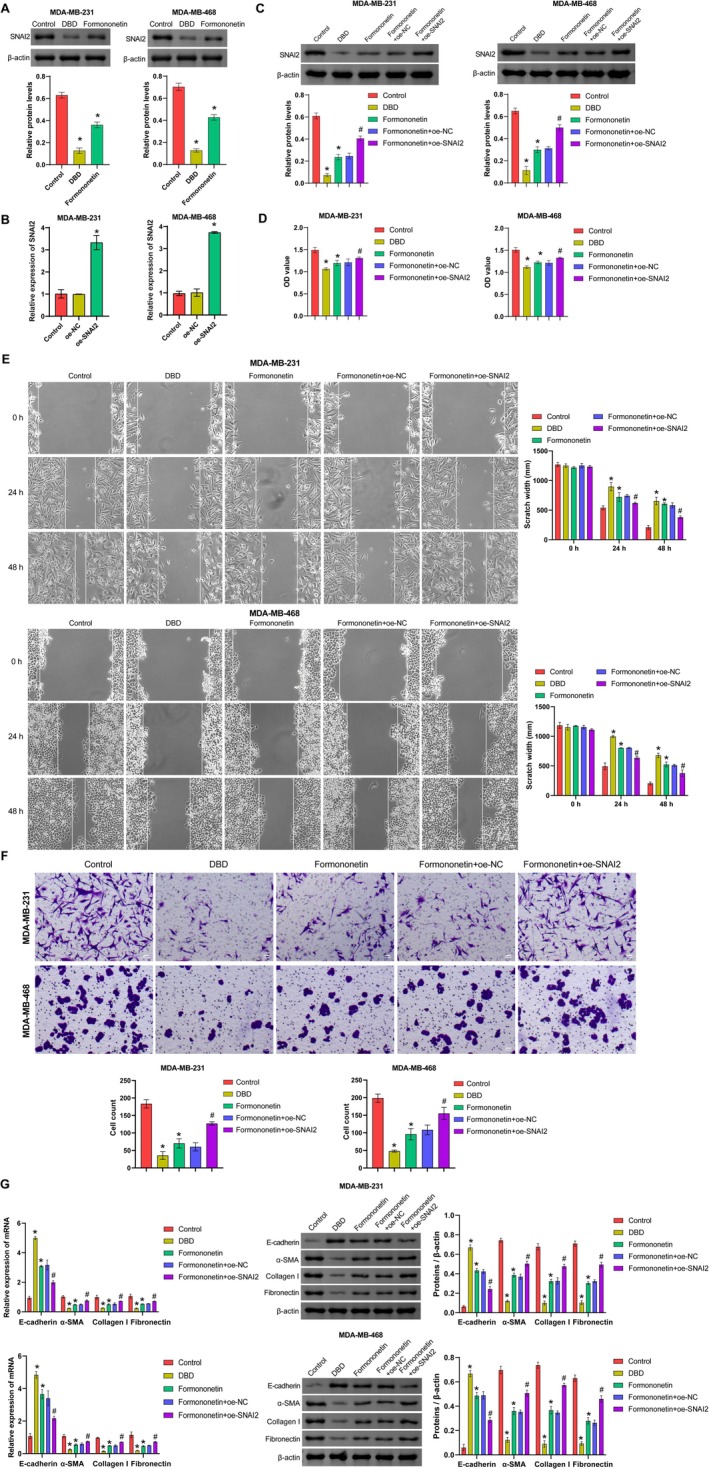
Formononetin inhibits cell proliferation, invasion, migration, and EMT by regulating SNAI2. (A) The effects of DBD and Formononetin on SNAI2 protein expression were detected by Western Blot. (B) The overexpression efficiency of oe‐SNAI2 was verified by qRT‐PCR. (C) The level of SNAI2 protein was detected by Western Blot. (D) Cell viability of TNBC cells was assessed by CCK‐8. (E) The migration of TNBC cells was assessed by the wound healing assay. (F) The invasion of TNBC cells was assessed by Transwell assay. (G) Expression of E‐cadherin, a‐SMA, Collagen I, and Fibronectin. Data are presented as mean ± SD, *n* = 3. *p < 0.05 vs. the Control group. # *p* < 0.05 vs. the Formononetin + oe‐NC group. Control group: untreated TNBC cells. DBD group: TNBC cells were treated with DBD. Formononetin group: TNBC cells were treated with Formononetin. Formononetin + oe‐NC group: TNBC cells were treated with Formononetin and then transfected with oe‐NC. Formononetin + oe‐SNAI2 group: TNBC cells were treated with Formononetin and then transfected with oe‐SNAI2.

To investigate the roles of DBD and Formononetin in TNBC, we initially conducted concentration screening experiments. CCK8 assays revealed that 1 mg/mL DBD and 80 μmol/L Formononetin significantly inhibited TNBC cell viability (Figure S1A,B). Furthermore, we found that 0–80 μmol/L Formononetin had no significant effect on the cell viability of normal MCF‐10A cells (Figure S2A), and the treatment with Formononetin did not cause any obvious pathological damage to the liver and kidney tissues of normal mice (Figure S2B). Consequently, we selected 1 mg/mL DBD and 80 μmol/L Formononetin to treat TNBC cells. Analysis of SNAI2 expression demonstrated that both 1 mg/mL DBD and 80 μmol/L Formononetin inhibited SNAI2 protein expression (Figure 5A).

Next, to explore whether Formononetin, the active component of DBD, exerts its effects through regulating SNAI2, we constructed an oe‐SNAI2 overexpression vector (Figure 5B) and performed functional loss‐of‐function experiments. Briefly, TNBC cells were treated with either Formononetin or DBD as single agents for 48 h, or transfected with SNAI2 overexpression plasmids following Formononetin treatment. As shown in Figure 5C, compared with the Formononetin + oe‐NC group, SNAI2 overexpression significantly reversed Formononetin‐induced inhibition of SNAI2 protein.

Cellular function analysis revealed that SNAI2 overexpression significantly reversed the inhibitory effects of Formononetin on TNBC cell proliferation and migration (Figure 5D–F). Furthermore, SNAI2 overexpression significantly reversed Formononetin's effects on the expression of a‐SMA, Collagen I, Fibronectin, and E‐cadherin (Figure 5G). These results suggest that Formononetin inhibits the malignant behavior of TNBC cells by targeting and suppressing SNAI2.

## 4 Discussion

The TCM perspective posits that the etiology of BC is attributable to liver and spleen dyfunction and Qi (vital energy) stagnation, which ultimately results in Qi depression (Guo et al. 2021, 2022). A multitude of studies have demonstrated the beneficial effects of TCM and its active ingredients on Qi and blood (Li et al. 2022), which can hinder the expansion and metastasis of TNBC through various mechanisms (Yang et al. 2021). In this study, the “Formononetin‐gene‐TNBC” network and HPLC‐MS were employed to identify Formononetin as a key component of DBD targeting TNBC. The experimental findings indicated that Formononetin can inhibit the malignant activity of TNBC tumors by targeting SNAI2. DBD's antitumor activities were systematically identified in this study, and robust evidence was provided for its use in anti‐TNBC.

Previous reports have demonstrated that DBD exhibits significant antitumor activity (Chen et al. 2016; Zhang et al. 2024). Consistent with these findings, our animal studies revealed that oral administration of DBD inhibited the growth and expansion of TNBC xenografts in nude mice. Furthermore, based on mass spectrometry identification and network pharmacology analysis, we identified Formononetin as one of the key active components of DBD. Although consistent with previous reports (Feng et al. 2021), the current HPLC‐MS analysis lacks absolute quantitative results. Future work should further clarify its absolute content in DBD. In vitro studies showed that treatment with DBD and Formononetin inhibited TNBC cell proliferation, invasion, migration, and EMT. Previous research indicated that the combination of DBD with doxorubicin promoted ferroptosis in TNBC cells, thereby inhibiting tumor progression (Gong et al. 2024). Formononetin suppressed the BACH1/p53 axis, thereby inhibiting TNBC tumor progression and metastasis (Li, Zhu, et al. 2023). Formononetin treatment enhanced the drug sensitivity of TNBC to Taxol and inhibited tumor growth (Li et al. 2021). These findings indicate that our research results are consistent with previously reported functions of DBD and Formononetin.

Previous studies have demonstrated that inhibition of SNAI2 expression mediated by the E3 ubiquitin ligase ASB13 can impede the metastasis of BC (H. Fan et al. 2020). There is an association between high SNAI1 expression and an increased tendency for migration, invasion, and tumorigenesis in TNBC (Ma et al. 2024; Singh et al. 2021). The targeted inhibition of SNAI1 has an anti‐TNBC effect (Fang et al. 2024). Our results of molecular docking analysis and DARTS indicated a binding between Formononetin and the core target SNAI2. Laboratory studies revealed that Formononetin inhibits the aggressive behavior of TNBC cells by focusing on SNAI*2*. This indicates that DBD or Formononetin could potentially be utilized in the future to lower SNAI2 expression, thus hindering the growth and spread of TNBC. Despite the promising results obtained in this study, there is a lack of positive controls at the cellular level in the studies, and currently, there is a lack of relevant clinical evidence. Further research is needed to assess DBD and Formononetin in anti‐TNBC. Furthermore, although our network pharmacology analysis also identified several other potential core targets, given that SNAI2 plays a crucial role in EMT and cancer metastasis, we chose SNAI2 for further research. The effects of the other predicted targets need to be further explored in future studies.

In conclusion, the key component of DBD, Formononetin, may inhibit the malignant activity of TNBC cells by reducing the expression of the transcription factor SNAI2, thereby exerting antitumor and antimetastasis effects. Our research results have better understood the key components and molecular mechanisms of DBD in treating TNBC. The inhibitory effect of DBD on TNBC is at least partially attributed to the regulation of SNAI2 by Formononetin. It is worth noting that the mechanism of action of traditional TCM typically exhibits characteristics of multi‐component and multi‐target synergy (Li, Liu, et al. 2023). Although this study has revealed the core role of Formononetin, other components in DBD (such as calycosin, ferulic acid, and Astragaloside IV) may enhance the bioavailability of Formononetin by regulating complementary signaling pathways, and produce a synergistic effect with it. It is necessary to systematically verify the interaction patterns among multiple components and explore the synergistic effects of other DBD components with Formononetin in the future, which may enhance its antitumor efficacy. In summary, DBD, as a multi‐target intervention TCM, shows potential advantages in the treatment of TNBC and is worthy of further development.

## Author Contributions

**Kui Li:** data curation (lead), formal analysis (lead), investigation (supporting), methodology (supporting), software (equal), validation (lead), visualization (lead), writing – original draft (lead). **Ting Zhou:** data curation (equal), formal analysis (equal), investigation (lead), methodology (lead), validation (equal), visualization (equal), writing – review and editing (equal). **Ruoxia Wu:** data curation (equal), investigation (equal), methodology (equal), software (equal), validation (equal), visualization (equal), writing – review and editing (equal). **Jiaqing Xiong:** data curation (equal), funding acquisition (lead), investigation (equal), methodology (equal), software (equal), validation (equal), visualization (equal), writing – original draft (equal), writing – review and editing (lead).

## Funding

The study was supported by the National Natural Science Foundation (No. 82205228) and Key Research Project of the Education Department of Hunan Province (No. 24A0251).

## Conflicts of Interest

The authors declare no conflicts of interest.

## Supporting information


**Figure S1:** Effects of DBD and active ingredient Formononetin on cytotoxicity. (A and B) CCK8 assay was used to detect the effect of DBD and Formononetin on TNBC cell cytotoxicity. Data are presented as mean ± SD, *n* = 3. * indicates a comparison with the 0 mg/mL or 0 μmol/L group, *p* < 0.05.

**Figure S2:** Safety assessment of Formononetin in both in vitro and in vivo studies. (A) CCK8 assay was used to detect the effect of Formononetin on MCF‐10A cell viability, *n* = 3. (B) Histopathological morphology of the liver and kidney tissues of the mice. Data are presented as mean ± SD, *n* = 6. * indicates a comparison with the 0 μmol/L or Sham group, *p* < 0.05.

## Data Availability

The data that support the findings of this study are available from the corresponding author, Jiaqing Xiong, upon reasonable request.

## References

[fsn371286-bib-0001] Arnold, M. , E. Morgan , H. Rumgay , et al. 2022. “Current and Future Burden of Breast Cancer: Global Statistics for 2020 and 2040.” Breast 66: 15–23. 10.1016/j.breast.2022.08.010. 36084384 PMC9465273

[fsn371286-bib-0002] Bao, X. , W. Li , R. Jia , D. Meng , H. Zhang , and L. Xia . 2023. “Molecular Mechanism of Ferulic Acid and Its Derivatives in Tumor Progression.” Pharmacological Reports 75, no. 4: 891–906. 10.1007/s43440-023-00494-0. 37202657 PMC10374777

[fsn371286-bib-0003] Chen, S. T. , T. Y. Lee , T. H. Tsai , et al. 2016. “The Traditional Chinese Medicine DangguiBuxue Tang Sensitizes Colorectal Cancer Cells to Chemoradiotherapy.” Molecules 21, no. 12: 1677. 10.3390/molecules21121677. 27929437 PMC6273051

[fsn371286-bib-0004] Fan, H. , X. Wang , W. Li , et al. 2020. “ASB13 Inhibits Breast Cancer Metastasis Through Promoting SNAI2 Degradation and Relieving Its Transcriptional Repression of YAP.” Genes & Development 34, no. 19–20: 1359–1372. 10.1101/gad.339796.120. 32943576 PMC7528707

[fsn371286-bib-0005] Fan, L. , H. Lei , S. Zhang , et al. 2024. “Erratum: Non‐Canonical Signaling Pathway of SNAI2 Induces EMT in Ovarian Cancer Cells by Suppressing miR‐222‐3p Transcription and Upregulating PDCD10: Erratum.” Theranostics 14, no. 3: 1098. 10.7150/thno.92973. 38250035 PMC10797293

[fsn371286-bib-0006] Fang, Y. , Y. Wang , H. Ma , et al. 2024. “TFAP2A Downregulation Mediates Tumor‐Suppressive Effect of miR‐8072 in Triple‐Negative Breast Cancer via Inhibiting SNAI1 Transcription.” Breast Cancer Research 26, no. 1: 103. 10.1186/s13058-024-01858-x. 38890750 PMC11186287

[fsn371286-bib-0007] Feng, S. H. , B. Zhao , X. Zhan , R. Motanyane , S. M. Wang , and A. Li . 2021. “Danggui Buxue Decoction in the Treatment of Metastatic Colon Cancer: Network Pharmacology Analysis and Experimental Validation.” Drug Design, Development and Therapy 15: 705–720. 10.2147/dddt.S293046. 33658761 PMC7917330

[fsn371286-bib-0008] Garrido‐Castro, A. C. , N. U. Lin , and K. Polyak . 2019. “Insights Into Molecular Classifications of Triple‐Negative Breast Cancer: Improving Patient Selection for Treatment.” Cancer Discovery 9, no. 2: 176–198. 10.1158/2159-8290.Cd-18-1177. 30679171 PMC6387871

[fsn371286-bib-0009] Gong, G. , K. Ganesan , Y. Liu , et al. 2024. “Danggui Buxue Tang Improves Therapeutic Efficacy of Doxorubicin in Triple Negative Breast Cancer via Ferroptosis.” Journal of Ethnopharmacology 323: 117655. 10.1016/j.jep.2023.117655. 38158099

[fsn371286-bib-0010] Grasset, E. M. , M. Dunworth , G. Sharma , et al. 2022. “Triple‐Negative Breast Cancer Metastasis Involves Complex Epithelial‐Mesenchymal Transition Dynamics and Requires Vimentin.” Science Translational Medicine 14, no. 656: eabn7571. 10.1126/scitranslmed.abn7571. 35921474 PMC9801390

[fsn371286-bib-0011] Guo, Q. , Q. Chen , C. C. Xue , A. L. Zhang , and M. E. Coyle . 2021. “Chinese Medicine Syndromes and Stages of Early Breast Cancer: Hierarchical Cluster Analysis and Implication for Clinical Practice.” Journal of Alternative and Complementary Medicine 27, no. 11: 904–914. 10.1089/acm.2021.0055. 34076505

[fsn371286-bib-0012] Guo, Q. , M. E. Coyle , A. L. Zhang , et al. 2022. “Chinese Medicine Syndrome Differentiation for Early Breast Cancer: A Multicenter Prospective Clinical Study.” Frontiers in Oncology 12: 914805. 10.3389/fonc.2022.914805. 35875101 PMC9300931

[fsn371286-bib-0013] Jiang, L. , L. Chen , W. Li , and R. Wang . 2023. “Role of Danggui Buxue Decoction for the Prevention and Treatment of Cardiovascular and Pulmonary Diseases.” Zhong Nan Da Xue Xue Bao. Yi Xue Ban 48, no. 10: 1479–1493. 10.11817/j.issn.1672-7347.2023.230198. 38432878 PMC10929893

[fsn371286-bib-0014] Jiang, T. , L. Xie , S. Zhou , et al. 2022. “Metformin and Histone Deacetylase Inhibitor Based Anti‐Inflammatory Nanoplatform for Epithelial‐Mesenchymal Transition Suppression and Metastatic Tumor Treatment.” Journal of Nanobiotechnology 20, no. 1: 394. 10.1186/s12951-022-01592-6. 36045429 PMC9429706

[fsn371286-bib-0015] Jiang, Z. , L. Pei , Y. Xie , et al. 2021. “Ruyiping Formula Inhibits Metastasis via the microRNA‐134‐SLUG Axis in Breast Cancer.” BMC Complementary Medicine and Therapies 21, no. 1: 191. 10.1186/s12906-021-03365-4. 34225726 PMC8258945

[fsn371286-bib-0016] Leon‐Ferre, R. A. , and M. P. Goetz . 2023. “Advances in Systemic Therapies for Triple Negative Breast Cancer.” BMJ 381: e071674. 10.1136/bmj-2022-071674. 37253507

[fsn371286-bib-0017] Li, C. , F. Zhu , C. Xu , et al. 2019. “Dangguibuxue Decoction Abolishes Abnormal Accumulation of Erythroid Progenitor Cells Induced by Melanoma.” Journal of Ethnopharmacology 242: 112035. 10.1016/j.jep.2019.112035. 31226383

[fsn371286-bib-0018] Li, J. W. , Q. M. Deng , J. L. Zhu , et al. 2023. “Methylation of ESR1 Promoter Induced by SNAI2‐DNMT3B Complex Promotes Epithelial‐Mesenchymal Transition and Correlates With Poor Prognosis in ERα‐Positive Breast Cancers.” MedComm 4, no. 6: e403. 10.1002/mco2.403. 37881785 PMC10594044

[fsn371286-bib-0019] Li, M. , T. Guo , J. Lin , et al. 2022. “Curcumin Inhibits the Invasion and Metastasis of Triple Negative Breast Cancer via Hedgehog/Gli1 Signaling Pathway.” Journal of Ethnopharmacology 283: 114689. 10.1016/j.jep.2021.114689. 34592340

[fsn371286-bib-0020] Li, S. , L. Zhu , Y. He , and T. Sun . 2023. “Formononetin Enhances the Chemosensitivity of Triple Negative Breast Cancer via BTB Domain and CNC Homolog 1‐Mediated Mitophagy Pathways.” Acta Biochimica Polonica 70, no. 3: 533–539. 10.18388/abp.2020_6466. 37672716

[fsn371286-bib-0021] Li, T. , S. Zhang , F. Chen , et al. 2021. “Formononetin Ameliorates the Drug Resistance of Taxol Resistant Triple Negative Breast Cancer by Inhibiting Autophagy.” American Journal of Translational Research 13, no. 2: 497–514. 33594306 PMC7868832

[fsn371286-bib-0022] Li, W. , M. Shen , Y. Z. Jiang , et al. 2020. “Deubiquitinase USP20 Promotes Breast Cancer Metastasis by Stabilizing SNAI2.” Genes & Development 34, no. 19–20: 1310–1315. 10.1101/gad.339804.120. 32943575 PMC7528704

[fsn371286-bib-0023] Li, X. , Z. Liu , J. Liao , Q. Chen , X. Lu , and X. Fan . 2023. “Network Pharmacology Approaches for Research of Traditional Chinese Medicines.” Chinese Journal of Natural Medicines 21, no. 5: 323–332. 10.1016/s1875-5364(23)60429-7. 37245871

[fsn371286-bib-0024] Liu, Z. , Z. Sun , D. Zhang , et al. 2020. “Paris Polyphylla Ethanol Extract Induces G2/M Arrest and Suppresses Migration and Invasion in Bladder Cancer.” Translational Cancer Research 9, no. 10: 5994–6004. 10.21037/tcr-20-1512. 35117211 PMC8797771

[fsn371286-bib-0025] Ma, X. , R. Wan , Y. Wen , T. Liu , Y. Song , and Y. Zhu . 2024. “Deubiquitinating Enzyme OTUD4 Regulates Metastasis in Triple‐Negative Breast Cancer by Stabilizing Snail1.” Experimental Cell Research 434, no. 1: 113864. 10.1016/j.yexcr.2023.113864. 38040050

[fsn371286-bib-0026] Nair, A. B. , and S. Jacob . 2016. “A Simple Practice Guide for Dose Conversion Between Animals and Human.” Journal of Basic and Clinical Pharmacy 7, no. 2: 27–31. 10.4103/0976-0105.177703. 27057123 PMC4804402

[fsn371286-bib-0027] Pan, B. , Y. Wang , C. Wu , et al. 2020. “A Mechanism of Action Study on Danggui Sini Decoction to Discover Its Therapeutic Effect on Gastric Cancer.” Frontiers in Pharmacology 11: 592903. 10.3389/fphar.2020.592903. 33505310 PMC7830678

[fsn371286-bib-0028] Qi, F. , L. Zhao , A. Zhou , et al. 2015. “The Advantages of Using Traditional Chinese Medicine as an Adjunctive Therapy in the Whole Course of Cancer Treatment Instead of Only Terminal Stage of Cancer.” Bioscience Trends 9, no. 1: 16–34. 10.5582/bst.2015.01019. 25787906

[fsn371286-bib-0029] Singh, D. , R. K. Deshmukh , and A. Das . 2021. “SNAI1‐Mediated Transcriptional Regulation of Epithelial‐To‐Mesenchymal Transition Genes in Breast Cancer Stem Cells.” Cellular Signalling 87: 110151. 10.1016/j.cellsig.2021.110151. 34537302

[fsn371286-bib-0030] Song, L. , X. Chen , L. Mi , et al. 2020. “Icariin‐Induced Inhibition of SIRT6/NF‐κB Triggers Redox Mediated Apoptosis and Enhances Anti‐Tumor Immunity in Triple‐Negative Breast Cancer.” Cancer Science 111, no. 11: 4242–4256. 10.1111/cas.14648. 32926492 PMC7648025

[fsn371286-bib-0031] Sung, H. , J. Ferlay , R. L. Siegel , et al. 2021. “Global Cancer Statistics 2020: GLOBOCAN Estimates of Incidence and Mortality Worldwide for 36 Cancers in 185 Countries.” CA: A Cancer Journal for Clinicians 71, no. 3: 209–249. 10.3322/caac.21660. 33538338

[fsn371286-bib-0032] Verma, A. , Y. M. Lam , Y. C. Leung , et al. 2019. “Combined Use of Arginase and Dichloroacetate Exhibits Anti‐Proliferative Effects in Triple Negative Breast Cancer Cells.” Journal of Pharmacy and Pharmacology 71, no. 3: 306–315. 10.1111/jphp.13033. 30362115

[fsn371286-bib-0033] Xu, X. , X. Y. Sun , M. Chang , et al. 2023. “Gemcitabine Enhances Pharmacokinetic Exposure of the Major Components of Danggui Buxue Decoction in Rat via the Promotion of Intestinal Permeability and Down‐Regulation of CYP3A for Combination Treatment of Non‐Small Cell Lung Cancer.” Pharmaceutical Biology 61, no. 1: 1298–1309. 10.1080/13880209.2023.2246500. 37606265 PMC10446811

[fsn371286-bib-0034] Yang, Z. , Q. Zhang , L. Yu , J. Zhu , Y. Cao , and X. Gao . 2021. “The Signaling Pathways and Targets of Traditional Chinese Medicine and Natural Medicine in Triple‐Negative Breast Cancer.” Journal of Ethnopharmacology 264: 113249. 10.1016/j.jep.2020.113249. 32810619

[fsn371286-bib-0035] Yuan, J. , Y. Liu , T. Zhang , et al. 2024. “Traditional Chinese Medicine for Breast Cancer Treatment: A Bibliometric and Visualization Analysis.” Pharmaceutical Biology 62, no. 1: 499–512. 10.1080/13880209.2024.2359105. 38813803 PMC11141317

[fsn371286-bib-0036] Zhang, Y. , Q. Kang , L. He , et al. 2024. “Exploring the Immunometabolic Potential of Danggui Buxue Decoction for the Treatment of IBD‐Related Colorectal Cancer.” Chinese Medicine 19, no. 1: 117. 10.1186/s13020-024-00978-y. 39210410 PMC11360867

[fsn371286-bib-0037] Zhu, D. , S. Li , L. Xu , et al. 2024. “Investigation of the Molecular Mechanism of Danggui Buxue Tang in Treating Lung Cancer Using Network Pharmacology and Molecular Docking Techniques.” Natural Product Research 39: 1–4. 10.1080/14786419.2024.2305660. 38403948

