# Supplementary figures and images for: Formononetin, a Key Component of Danggui Buxue Decoction, Inhibits Metastasis of Triple‐Negative Breast Cancer by Modulating SNAI2‐Driven EMT
Source: Food Sci Nutr. 2025 Dec 4;13(12):e71286. doi: 10.1002/fsn3.71286 (PMC12678057; doi:10.1002/fsn3.71286)

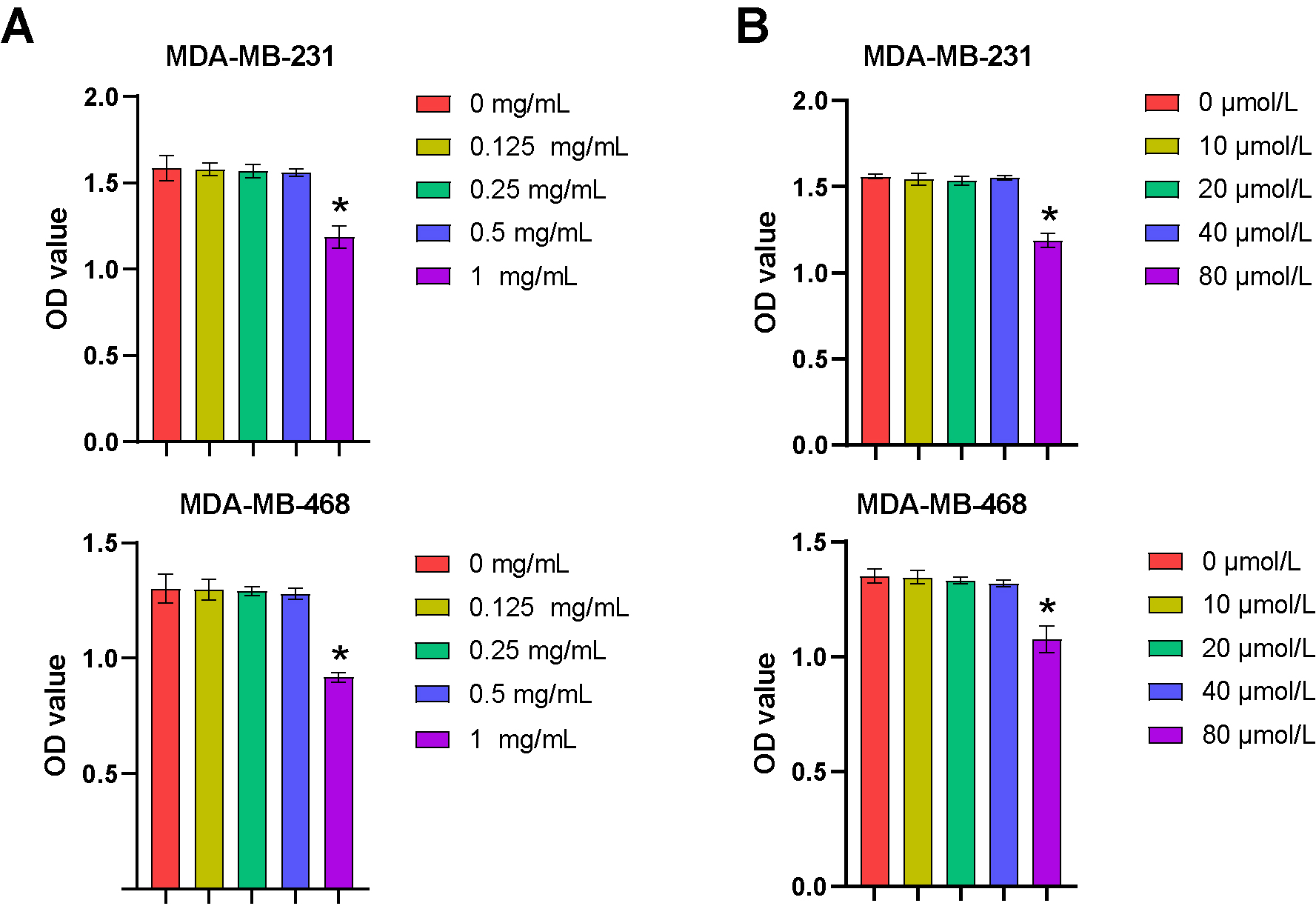

Supplement: Supplementary file 1 — Figure S1: Effects of DBD and active ingredient Formononetin on cytotoxicity. (A and B) CCK8 assay was used to detect the effect of DBD and Formononetin on TNBC cell cytotoxicity. Data are presented as mean ± SD, n = 3. * indicates a comparison with the 0 mg/mL or 0 μmol/L group, p < 0.05. [file FSN3-13-e71286-s001.jpg]

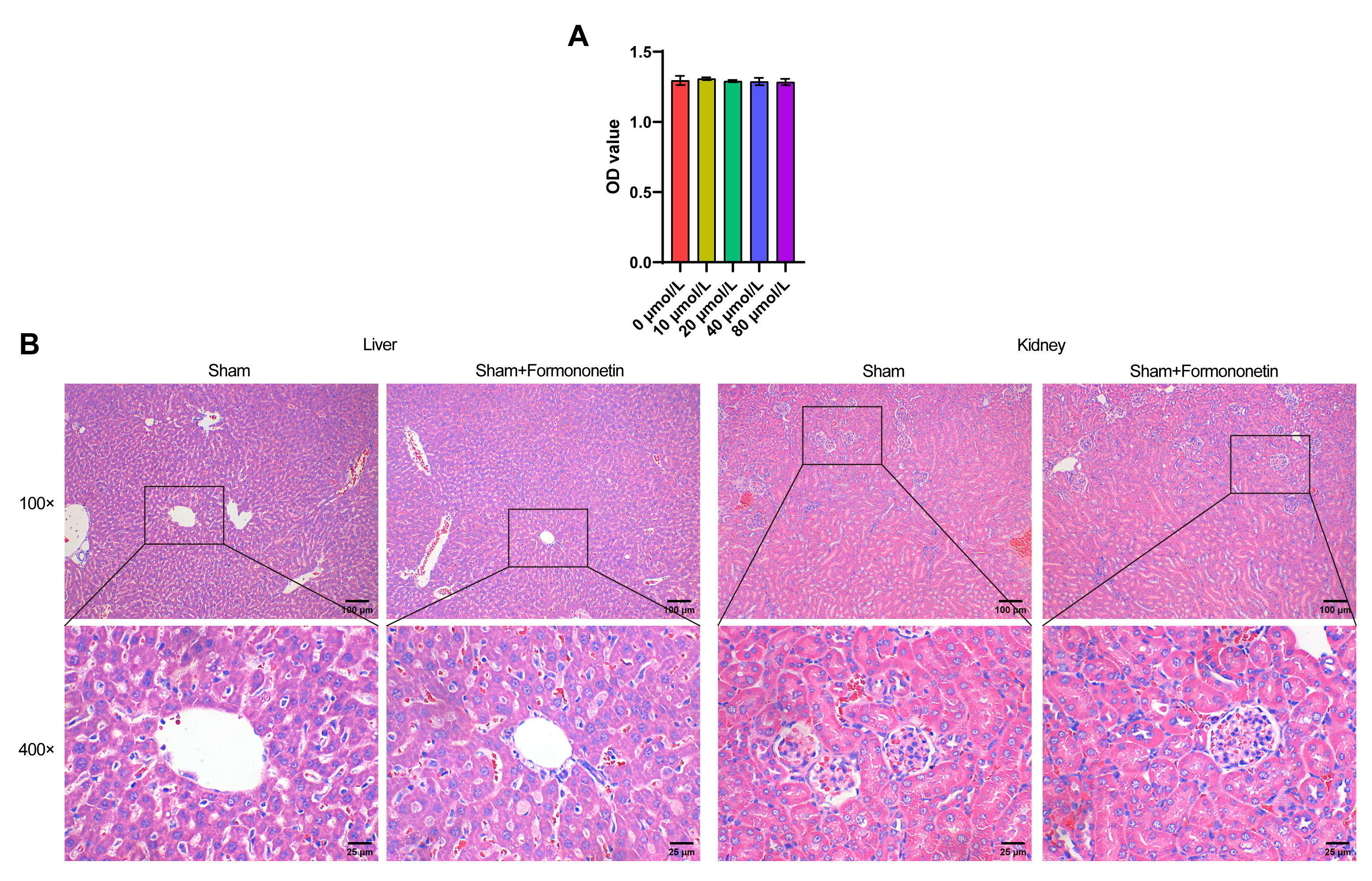

Supplement: Supplementary file 2 — Figure S2: Safety assessment of Formononetin in both in vitro and in vivo studies. (A) CCK8 assay was used to detect the effect of Formononetin on MCF‐10A cell viability, n = 3. (B) Histopathological morphology of the liver and kidney tissues of the mice. Data are presented as mean ± SD, n = 6. * indicates a comparison with the 0 μmol/L or Sham group, p < 0.05. [file FSN3-13-e71286-s002.jpg]
